# Correlation of endoplasmic reticulum stress patterns with the immune microenvironment in hepatocellular carcinoma: a prognostic signature analysis

**DOI:** 10.3389/fimmu.2023.1270774

**Published:** 2023-12-08

**Authors:** Ke Zhan, Xin Yang, Shuang Li, Yang Bai

**Affiliations:** ^1^ Department of Gastroenterology, The First Affiliated Hospital of Chongqing Medical University, Chongqing, China; ^2^ Department of Gastrointestinal Surgery, Jinshan Hospital, The First Affiliated Hospital of Chongqing Medical University, Chongqing, China; ^3^ Department of Respiratory and Critical Care Medicine, The First Affiliated Hospital of Chongqing Medical University, Chongqing, China

**Keywords:** hepatocellular carcinoma, endoplasmic reticulum stress, prognosis, immune microenvironment, gp6

## Abstract

**Backgrounds:**

The extended duration of endoplasmic reticulum stress (ERS) can impact the progression of hepatocellular carcinoma (HCC) and the efficacy of immunotherapies by interacting with immune cells that have infiltrated the tumor microenvironment (TME).

**Methods and results:**

The study utilized a training cohort of 364 HCC patients with complete information from The Cancer Genome Atlas Program (TCGA) database, and a validation cohort of 231 HCC patients from the International Cancer Genome Consortium (ICGC) database. The genes related to ERS exhibiting a strong correlation with overall survival (OS) were identified using univariate Cox regression analysis. A 13-gene predictive signature was then produced through the least absolute shrinkage and selection operator (LASSO) regression approach. The data revealed that the ERS-associated gene signature effectively stratified patients into high- or low-risk groups regarding OS in both the training and validation cohorts (P < 0.0001 and P = 0.00029, respectively). Using the multivariate method, it is still an independent prognostic factor in both the training and validation cohorts (P < 0.001 and P = 0.008, respectively). Moreover, several metabolic pathways were identified to be enriched among the 13 genes in the predictive signature. When the ERS-associated gene signature was combined with the tumor-node-metastasis (TNM) stage, the ERS nomogram performed better than either the gene signature or the TNM stage alone (C-index values: 0.731, 0.729, and 0.573, respectively). Further analysis revealed that patients in the high-risk group exhibited increased infiltration of immune cells. Additionally, GP6 was downregulated in HCC tissues among these signature genes (P < 0.05), which was related to poor OS.

**Conclusions:**

The data suggest that this novel ERS-associated gene signature could contribute to personalized cancer management for HCC. Moreover, targeting GP6 inhibition might be a potential method for HCC therapy.

## Introduction

Ranked as the sixth most diagnosed cancer and third most common reason for cancer-associated death worldwide, liver cancer shows a 5-year survival rate of merely 18% (1, 2). Hepatocellular carcinoma (HCC) accounts for ≥ 90% of primary liver cancer cases and usually develops in individuals with pre-existing chronic liver disease (3). The grade of the tumor largely determines the dismal prognosis of HCC at the point of initial diagnosis (4). The Barcelona Clinic Liver Cancer classification is widely utilized as a staging system for predicting HCC prognosis; however, it does not consider the impact of differentially expressed genes (DEGs) and functional pathways on disease progression (5). With the advent of microarray technology and bioinformatics, it has become increasingly clear that DEGs and functional pathways are essential in the tumorigenesis and development of HCC (6). Therefore, we hypothesized that combining genomic data with clinical and demographic characteristics could enhance the accuracy of prognostic prediction in HCC patients.

The unfolded protein response (UPR) activation was highlighted in the tumor progression of various types of cancer (7). UPR is a preserved adaptive mechanism employed by cells to manage endoplasmic reticulum stress (ERS) (8). Sustained ERS can trigger the inflammatory response through the UPR pathway, involving tumorigenesis, progression, metastasis, and chemoresistance in HCC (9). However, in some cases, ERS has also been associated with a more favorable prognosis (10). ERS-associated genes and signaling pathways might represent significant biomarkers for the prognostic prediction of HCC patients. Tumor immune microenvironment (TME) assumes a critical role in the onset and advancement of HCC, and is closely linked to the response or resistance to immunotherapies (11). Sustained ERS can regulate HCC progression by interacting with immune cells in the TME (12). In the study, we conducted Cox proportional hazard regression analysis on 59 ERS-associated genes in The Cancer Genome Atlas Liver Hepatocellular Carcinoma (TCGA-LIHC) cohort to analyze genes related to prognosis. The significant candidates were utilized to generate a gene signature risk score using the least absolute shrinkage and selection operator (LASSO) Cox regression approach, and this was subsequently validated in the independent International Cancer Genome Consortium (ICGC) Liver Cancer-RIKEN, Japan (LIRI-JP) cohort. A survival study was conducted to establish the degree to which the gene signature risk score contributed to patients’ overall survival (OS) rates. Patients were placed into high-risk or low-risk groups according to the average risk score. Gene Set Enrichment Analysis (GSEA) was used to analyze the differences in substantial signaling between the group considered high risk and the group considered low risk. A nomogram is constructed to incorporate the tumor-node-metastasis (TNM) stage and the prognostic gene signature to assess an individual’s likelihood of survival. This allows for the likelihood of individual survival to be calculated. Additionally, we validated GP6 downregulation in HCC tissues using quantitative reverse transcription polymerase chain reaction (qRT-PCR) and demonstrated that it was related to poor OS. These data suggest that this unique ERS-associated gene profile might benefit personalized cancer management in HCC and that GP6 inhibition could represent a promising new therapeutic target for HCC.

## Materials and methods

### Selection of ERS-associated genes

The term “endoplasmic reticulum stress” was used to search the GeneCards website for ERS-associated genes. ERS-associated genes were defined as genes having a relevance score greater than 7.

### Acquisition of HCC cohorts

The training cohort comprised gene expression profiles obtained from HCC cohorts retrieved from the TCGA website, while the validation cohort consisted of data obtained from the ICGC website. The gene expression profile was normalized by using the “VST” function that is part of the “DESeq2” R package. The TCGA database and the cBioPortal, respectively, were the sources of the clinical information and data on somatic mutations that were used for the TCGA-LIHC cohort. With the ICGC database, we gathered information on the clinical conditions and somatic mutations present in the ICGC LIRI-JP cohort. Also, the TCGA-LIHC data of simple nucleotide variation were produced from the TCGA database, and the gene copy number was collected from The University of California Santa Cruz (UCSC) database. Both of these databases were utilized, which are accessible from this location. The “Rcircos” R package was employed to depict the copy number variation (CNV) landscape of the ERS genes on human chromosomes.

### Identification and validation of the prognostic gene signature

Differential expression analysis of ERS-related genes was carried out using the “DEseq2” software included in R using the criterion of having a |logFC| value of more than 1 and an adjusted P-value of less than 0.05. The TCGA-LIHC dataset was subsequently used to perform a univariate Cox proportional hazard regression analysis to investigate ERS genes substantially connected to OS. The identified OS-related genes were utilized in conjunction with the LASSO Cox regression to generate a predictive multiple-gene signature via the “glmnet” R package (13, 14). A risk score formula was developed using the following equation: Risk score (based on mRNA expression) = sum of coefficients × mRNA expression levels. The HCC cohorts were sorted into two groups using the average risk score as the cutoff. The independent ICGC LIRI-JP cohorts served as a means of verifying the predictive gene signature.

### GSEA

We used GSEA version 4.0.3, which allowed us to analyze the potential biological processes or signaling pathways connected with the signature genes. We used pre-defined sets of genes found in the MsigDB (c2.cp.Kegg.v7.0.symbols.gmt). Patients were assigned to either a high-risk or a low-risk group based on their gene signature risk scores. The criteria of a nominal P-value of less than 0.05 and a false discovery rate of less than 0.25 were used to conclude on the significance of the normalized enrichment score.

### Evaluation of immune cell infiltration

The deconvolution method CIBERSORT converts was employed to translate the normalized gene expression matrix into a representation of the infiltrated immune cells (15). During the CIBERSORT computation, the number of distinct cell types in complicated tissue was assessed, and the CIBERSORT results were validated using fluorescence-activated cell sorting (FACS). For reference expression signature, LM22 was utilized with 1000 permutations. A P-value of 0.05 was considered a more accurate estimation of the composition of immune cells by CIBERSORT. The samples that satisfied the constraint were then used for further study. For each sample, all 22 kinds of immune cell fractions were to sum up to one. Each individual’s relative percentages of 22 subpopulations of immune cells were shown using a bar plot. The violent diagrams were constructed to represent variations in immune cell infiltration using the “ggplot2” R package. The “corrplot” R package generated a correlation heatmap depicting the relationship of all cell subpopulations. The “ggstatsplot” R package was utilized to carry out a Spearman correlation analysis between diagnostic biomarkers and infiltrated immune cells. The results were generated using the “ggplot2” R package.

### Patients and Specimens

With the patients’ consent, 9 matched HCC samples and paired adjacent nontumorous samples were obtained from individuals who underwent hepatectomy at the Second Affiliated Hospital of Chongqing Medical University between January 2018 and July 2019. Inclusion criteria for patient selection included a definitive diagnosis of HCC by a pathologist, surgical resection with histological confirmation of tumor-free margins, and no prior chemo- or radiotherapy. The freshly obtained specimens were immediately kept using liquid nitrogen until further processing.

### Cell culture

ATCC was contacted to acquire the human noncancerous hepatic cell line MIHA and five human HCC cell lines (Huh7, HepG2, SK-hep1, MHCC-97H, and MHCC-97L). Cells were grown in a humidified incubator with 5% CO2 at 37 degrees Celsius using DMEM (Gibco) containing 10% heat-inactivated FBS (Gibco), penicillin (100 U/mL) (Beyotime, Shanghai, China), and streptomycin (100 g/mL) (Beyotime, Shanghai, China).

### qRT-PCR

After utilizing the TRIzol Reagent (Life Technologies) to initially isolate total RNA from fresh specimens or cells, reverse transcription was carried out using the PrimeScript RT Reagent Kit (Takara). The first phase of the PCR amplification procedure is a denaturation step carried out at 95 degrees Celsius for 10 minutes. This is followed by 35 cycles of a two-step PCR carried out at 95 degrees Celsius for 14 seconds and 60 degrees Celsius for 1 minute. We used the 2^-ΔΔCt^ approach to determine the relative expression of genes after first using GAPDH to standardize the cycle times (Ct) of the genes of interest. SYBR Green I was used for the RT-qPCR that was performed (Takara). The following primer pairs were utilized: GP6, forward: 5’-TCCCGGCCATGAAGAGAAGT-3’ and reverse: 5’-TTACGTCCCCTCCTGACGAC-3’; CASQ2, forward: 5’-GGCAGAAGAGGGGCTTAATTT-3’ and reverse: 5’-GAAGACACCGGCTCATGGTAG-3’; GAPDH, forward: 5’-GATCATCAGCAATGCCTCCT-3’ and reverse: 5’- GAGTCCTTCCACGATACCAA-3’.

### Development of nomogram

According to multivariable analyses, factors with P < 0.05 were chosen for further nomogram creation. Subsequently, a nomogram was generated using the “survival” and “rms” packages in R, which incorporated the 13-ERS-related gene signature and TNM stage as quantitative predictors of clinical prognosis. Calibration curves were constructed to assess the agreement between predicted and actual survival, and the C-index, which ranged from 0.5 to 1.0, was generated to determine the model’s efficacy in prognostic prediction. The numbers 0.5 and 1.0 reflect a random chance and an outstanding ability to predict survival with the model.

### Statistical analysis

R, version 4.2.0, was used throughout each statistical analysis carried out. In order to determine whether or not there was a correlation between risk scores and clinical factors, either the 2 test or Fisher’s exact test was applied. The Wilcoxon matched-pairs test was used in order to make a comparison between the levels of GP6 and CASQ2 that were found in HCC tissues and the levels that were found in neighboring normal tissues. In order to investigate the connection between risk scores and OS, both univariate and multivariate versions of the Cox proportional hazard regression model were used. The one-way analysis of variance (ANOVA) was used to compare the levels of GP6 and CASQ2 expression in noncancerous liver cells to the levels of expression in five HCC cell lines. The Kaplan-Meier technique and the log-rank test were used to compare OS rates among the different experimental groups. In order to analyze the survival prediction’s specificity and sensitivity using the gene signature risk score, receiver operating characteristic (ROC) analysis was performed, and the area under the curve (AUC) was applied in order to investigate the accuracy of the prognosis. Where P was less than 0.05, statistical significance was assumed.

## Results

### Development of the prognostic gene signature

**Figure 1:**
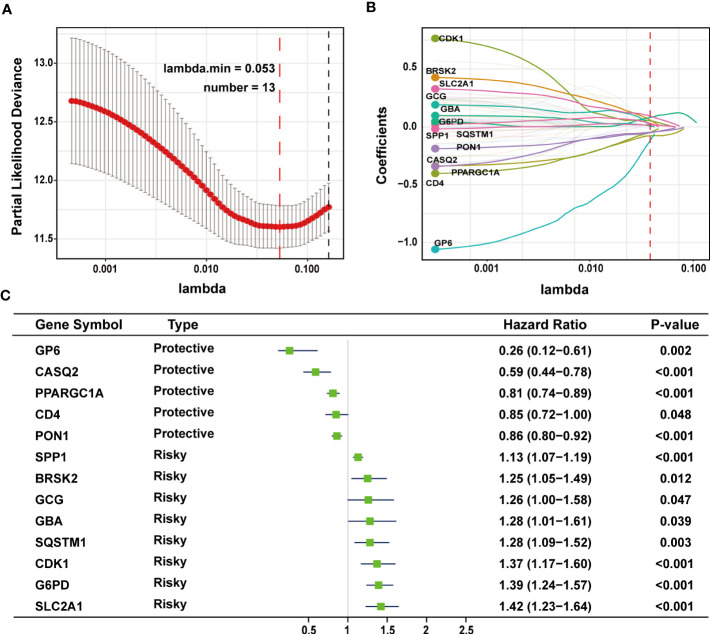
LASSO regression analysis was employed to develop the predictive gene signature. **(A)** A plot of the coefficient profile was made with the log (lambda) sequence. Choosing the ideal parameter (lambda) for the TCGA-LIHC LASSO model. **(B)** Profiles of the 13 genes’ LASSO coefficients from the TCGA-LIHC. **(C)** A forest plot representing the 13 genes linked to survival. LASSO, least absolute shrinkage and selection operator; TCGA-LIHC, The Cancer Genome Atlas Liver Hepatocellular Carcinoma.

**Figure 2:**
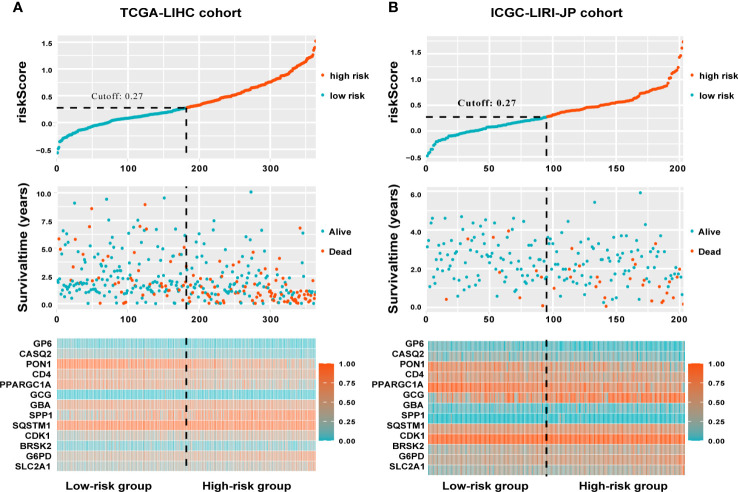
A visualization of the distributions of risk scores and survival status in the training **(A)** and validation **(B)** cohorts, as well as a heatmap of gene expression profiles of genes related to endoplasmic reticulum stress. Patients are dichotomized into low- and high-risk groups using the dotted line.

The TCGA-LIHC cohort contained 364 patients with comprehensive information, including gender, age, tumor grade, and TNM stage, whereas the ICGC LIRI-JP cohort had 231 individuals. **Table S1** shows the detailed baseline features of patients in both groups. We obtained 778 genes in ERS with a relevance score greater than 7 directly or indirectly from the GeneCards website. Next, because of the lack of expression in the TCGA-LIHC cohort, we excluded 17 genes. Lastly, we retrieved 761 ERS-associated genes from the TCGA-LIHC for gene expression profiles, of which 168 DEGs in HCC were found for future research on prognostic significance (**Figure S1**). 168 HCC DEGs were submitted to univariate Cox regression, with 59 ERS-associated genes identified to substantially correlate with OS in the TCGA-LIHC cohort (**Table S2**). The regression coefficients of these important genes were generated using LASSO COX regression (**Figure S2**). The model performed best when all 13 genes were included (**Figure 1A**). The regulatory roles of these 13 genes, primarily engaged in ERS, were reported in **Table 1**, and the lasso coefficient of the 13 genes was shown in **Figure 1B**. Five genes (GP6, CASQ2, PON1, CD4, PPARGC1A) with hazard ratios (HR) less than one (all P < 0.05) were considered protective, whereas eight genes (GCG, GBA, SPP1, SQSTM1, CDK1, BRSK2, G6PD, SLC2A1) with HR more than one (all P < 0.05) were considered dangerous (**Figure 1C**). The risk score for every individual was calculated as a linear combination of the level of each ERS-associated gene multiplied by its corresponding LASSO regression coefficient. The average risk score was considered a threshold to stratify individuals in high- or low-risk groups for prognostic prediction. **Figure 2** illustrates the distribution of the 13-ER-stress-gene-based risk scores, the survival time of the patient, and outcomes in the training and validation cohorts. The heat map suggested the eight risky genes were more abundantly expressed in the high-risk group, whereas the five protective genes were more abundantly detected in the low-risk group.

**Table 1:** Functions of genes in the prognostic gene signature.

NO.	Gene Symbol	Full name	Function	Relevance score	Risks coefficients
1	GP6	Glycoprotein VI Platelet	Involves in collagen-induced platelet adhesion and activation	7.38	-0.1194
2	CASQ2	Calsequestrin 2	Regulates the release of lumenal Ca(2+)	11.47	-0.0771
3	PON1	Paraoxonase 1	High-density-lipoprotein associated enzyme	11.69	-0.0507
4	CD4	CD4 Molecule	Cell differentiation antigen CD4	17.75	-0.0470
5	PPARGC1A	PPARG Coactivator 1 Alpha	Transcriptional coactivator for steroid receptors and nuclear receptors	8.64	-0.0466
6	GCG	Glucagon	Regulates blood glucose	8.89	0.0072
7	GBA	Glucosylceramidase Beta	Glucosylceramidase	8.27	0.0073
8	SPP1	Secreted Phosphoprotein 1	Osteopontin	17.19	0.0156
9	SQSTM1	Sequestosome 1	Autophagy receptor required for selective macroautophagy	12.27	0.0302
10	CDK1	Cyclin Dependent Kinase 1	Controls the eukaryotic cell cycle	8.13	0.0519
11	BRSK2	BR Serine/Threonine Kinase 2	Serine/threonine-protein kinase	11.12	0.0584
12	G6PD	Glucose-6-Phosphate Dehydrogenase	Catalyzes the first and rate-limiting step of the oxidative branch within the pentose phosphate pathway/shunt	9.8	0.0808
13	SLC2A1	Solute Carrier Family 2 Member 1	Facilitative glucose transporter	7.75	0.0925

### Validation of the prognostic gene signature

**Figure 3:**
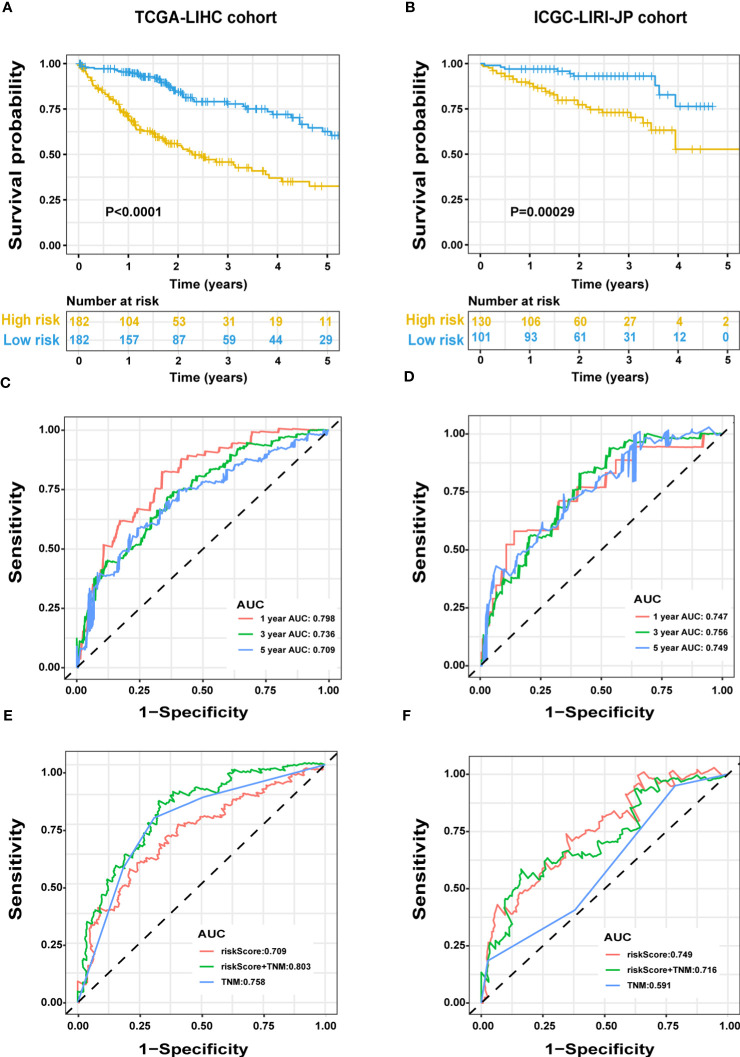
Predictive performance of the ERS-associated gene signature in HCC. OS in the training **(A)** and validation **(B)** populations shown by Kaplan-Meier plots. Time-dependent ROC curves based on the gene signature in the training **(C)** and validation **(D)** cohorts, with computed AUCs at 1-, 3-, and 5-year OS. ROC analysis of the OS’s sensitivity and specificity in the training **(E)** and validation **(F)** cohorts for the TNM stage and risk score combination. ERS, endoplasmic reticulum stress; HCC, hepatocellular carcinoma; OS, overall survival; ROC, receiver operating characteristic; AUC, area under the curve; TNM, tumor-node-metastasis.

We evaluated the predictive capacity of the 13-gene signature in the TCGA-LIHC and ICGC LIRI-JP cohorts. The results showed that the HCC patients with low-risk scores exhibited remarkably longer survival than those with high-risk scores in both the training (P < 0.0001) and validation groups (P = 0.00029) (**Figures 3A, B**). A time-dependent ROC curve was further generated for OS at various periods to assess the signature’s predictive capabilities, and the AUC values for 1-, 3-, and 5-year OS were 0.798, 0.736, and 0.709, respectively, in the TCGA-LIHC group (**Figure 3C**), and 0.747, 0.756, and 0.749, respectively, in the ICGC LIRI-JP cohort (**Figure 3D**). Moreover, combining the TNM stage and risk score could enhance prognostic accuracy in the TCGA-LIHC cohort (**Figure 3E**) compared to the TNM stage or risk score alone. The risk score in the ICGC LIRI-JP cohort exhibited an enhanced predictive value than the TNM stage and the combination of both (**Figure 3F**).

**Figure 4:**
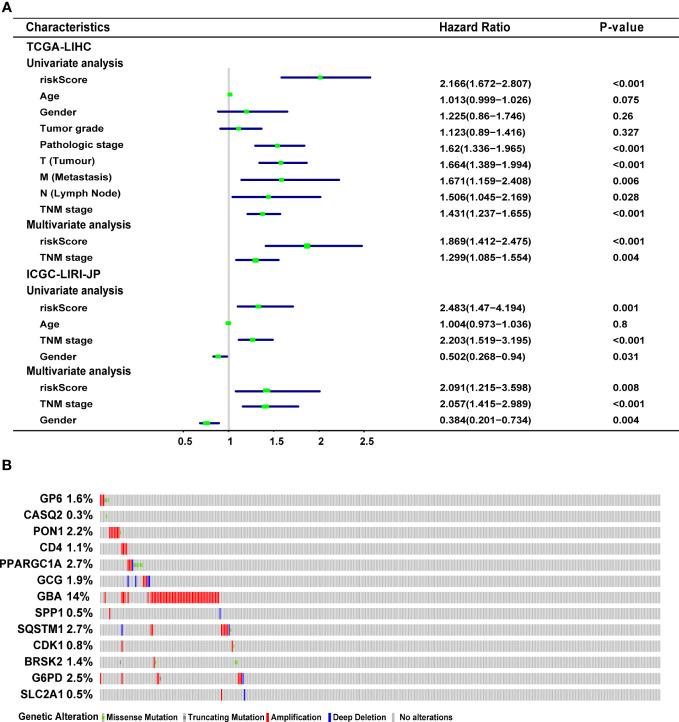
Characteristics of the ERS-associated gene signature. **(A)** Univariate and multivariate Cox regression analyses the relationship between the signature and clinical characteristics in the training and validation cohorts. **(B)** Genetic modification of the 13 genes in the TCGA-LIHC cohort (TCGA, Provisional). ERS, endoplasmic reticulum stress; TCGA-LIHC, The Cancer Genome Atlas Liver Hepatocellular Carcinoma.

**Figure 5:**
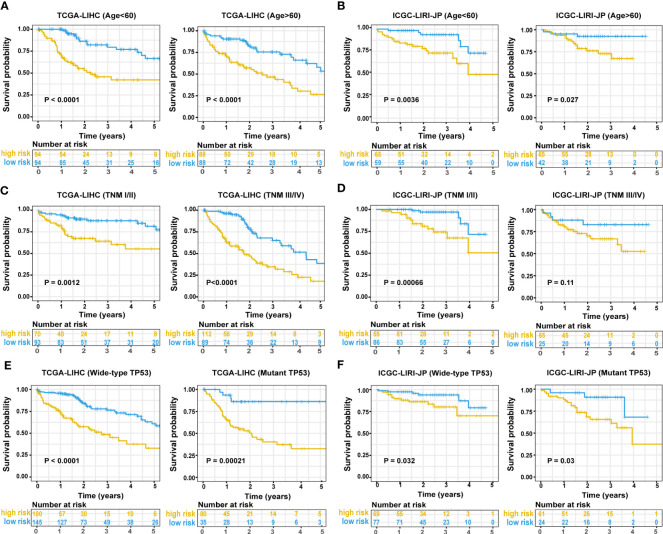
Predictive performance of the ERS-associated gene signature in subgroups. A Kaplan-Meier plot shows OS in subgroups based on age in training **(A)** and validation cohorts **(B)**. A Kaplan-Meier plot shows OS in subgroups based on tumor stage in the training **(C)** and validation cohorts **(D)**. A Kaplan-Meier plot shows OS in subgroups based on statuses of TP53 mutation in the training **(E)** and validation cohorts **(F)**. ERS, endoplasmic reticulum stress; OS, overall survival.

In the TCGA-LIHC cohort, a higher risk score was notably related to a higher TNM stage and advanced grade of tumor. In the validation cohort, a higher risk score was exclusively related to a higher TNM stage (**Table S1**). Univariate Cox regression analysis showed that the risk score was remarkably related to poor prognosis in both the training (HR = 2.166, 95% CI = 1.672-2.807, P < 0.001) and validation (HR = 2.483, 95% CI = 1.470-4.194, P = 0.001) cohorts (**Figure 4A**). TNM stage (HR = 1.431, 95% CI = 1.237-1.655, P = 0.004) and pathologic stage (HR = 1.62, 95% CI = 1.336-1.994, P < 0.001) were strong predictors of survival in the training cohort, while TNM stage (HR = 2.203, 95% CI = 1.519-3.195, P < 0.001) and gender (HR = 0.502, 95% CI = 0.268-0.94, P = 0.031) were significant predictors in the validation cohort (**Figure 4A**). In the training cohort, both the signature-based risk score (HR = 1.869, 95% CI = 1.412-2.475, P = 0.001) and the TNM stage (HR = 1.299, 95% CI = 1.085-1.554, P = 0.004) were independent predictors of OS using Cox multivariate regression analysis (**Figure 4A**). In the validation cohort, the signature-based risk score (HR = 2.091, 95% CI = 1.215-3.598, P = 0.008), TNM stage (HR = 2.057, 95% CI = 1.415-2.989, P = 0.001), and gender (HR = 0.384, 95% CI = 0.201-0.734, P = 0.004) were all independent predictors of OS using Cox multivariate regression analysis (**Figure 4A**). We investigated the genetic changes in these risk-associated genes to better understand their role in HCC (http://www.cbioportal.org). In this study, we utilized two data sets for TCGA-LIHC: the Provisional data set (366 samples) and the PanCancer Atlas data set (353 samples). Only samples that had both mutation and CNV data were included in the analyses. Genes of interest are changed in 75 (21%) of 353 PanCancer Atlas questioned samples (**Figure S3**) but not in 95 (26%) of 366 Provisional samples (**Figure 4B**). The frequent genetic changes show that these genes are essential during the formation and development of HCC. We performed a Kaplan-Meier analysis of survival in HCC patients by using the TNM stage, the status of TP53 mutation, and age. The ERS-associated gene signature accurately distinguished OS rates in different subgroups in the TCGA-LIHC cohort (all P < 0.05) (**Figures 5A, C, E**). In the ICGC LIRI-JP cohort, patients with low-risk scores exhibited remarkably improved OS than those with high-risk scores at the age of > 60 years (P = 0.027), ≤ 60 years (P = 0.0036), wild-type TP53 (P = 0.032), and mutant TP53 (P = 0.03) (**Figures 5B, F**). However, in the TNM stages III+IV subgroup, no significant difference was observed in OS between the high- and low-risk group (P = 0.11), whereas in the TNM stages I+II subgroup, patients in the high-risk group exhibited remarkably worse OS than those in the low-risk group (P = 0.00066) (**Figure 5D**). This prognostic signature may enhance the sensitivity and specificity of the standard TNM model, resulting in a benefit that could potentially aid in clinical decision-making.

### Identifying signaling pathways related to the predictive gene signature

**Figure 6:**
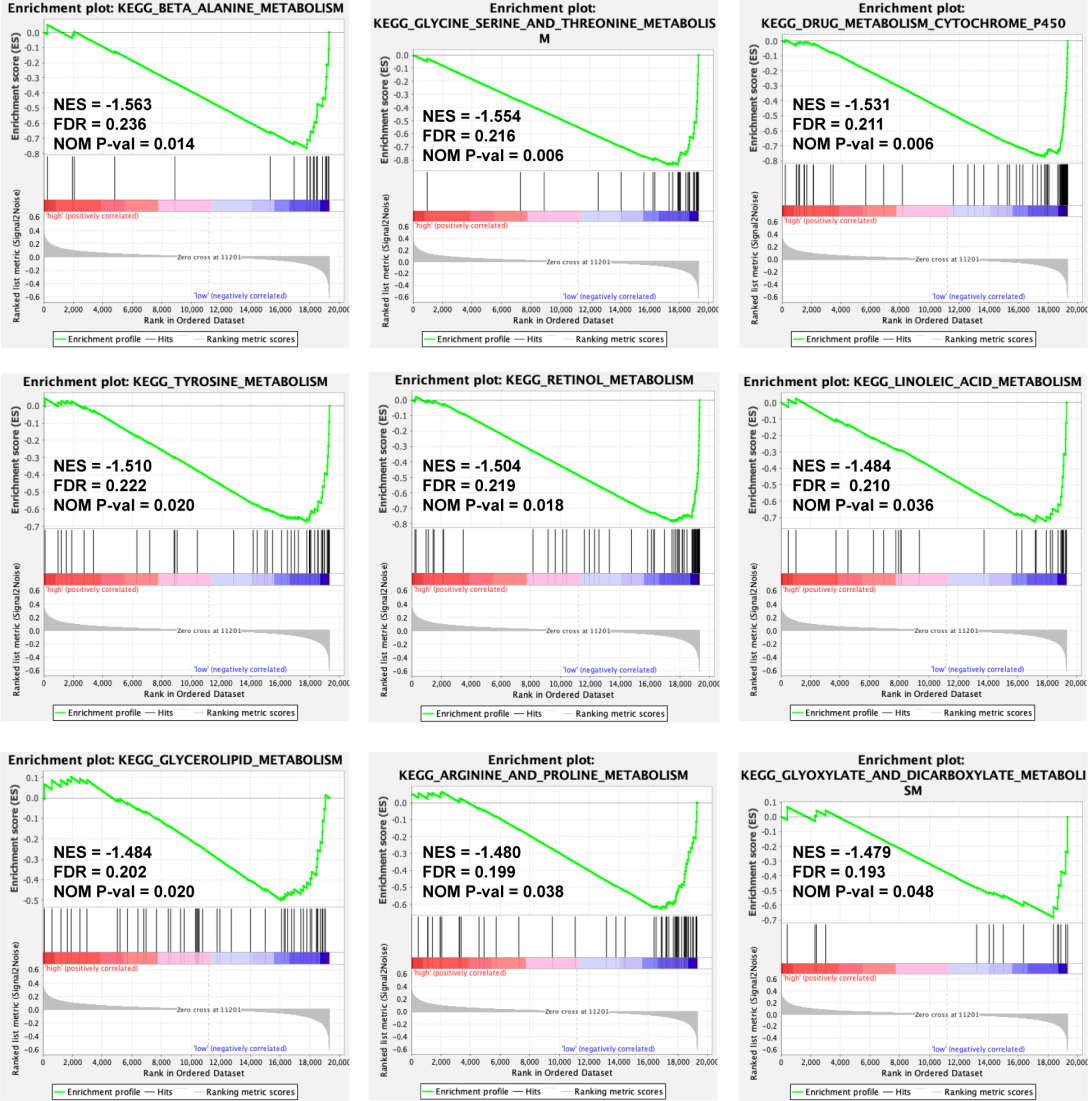
A GSEA plot showing KEGG pathways that are commonly enriched in the TCGA-LIHC cohort. GSEA, Gene Set Enrichment Analysis; KEGG, Kyoto Encyclopedia of Genes and Genomes; TCGA-LIHC, The Cancer Genome Atlas Liver Hepatocellular Carcinoma; NES, Normalized Enrichment Score; FDR, False Discovery Rate; NOM P-val, normal P value.

To discover the molecular pathways underlying the ERS-associated gene signature, GSEA was used to compare high- and low-risk groups in the TCGA-LIHC and ICGC LIRI-JP cohorts. In the TCGA-LIHC and ICGC LIRI-JP cohorts, 23 and 16 Kyoto Encyclopedia of Genes and Genomes (KEGG) pathways, respectively, were enriched (**Table S3**). **Figure 6** depicts the KEGG pathways that are commonly enriched in both cohorts. The cell cycle, spliceosome, and homologous recombination were shown to be strongly related to the high-risk group. Meanwhile, the low-risk group was related to drug metabolism cytochrome p450, insulin signaling, adipocytokine signaling, renin-angiotensin system, retinol metabolism, butanoate metabolism, metabolism of glyoxylate and dicarboxylate or fatty acid, amino acid metabolic pathways, and lipid metabolic pathways.

### The landscape of genetic variations of the signature genes in HCC

**Figure 7:**
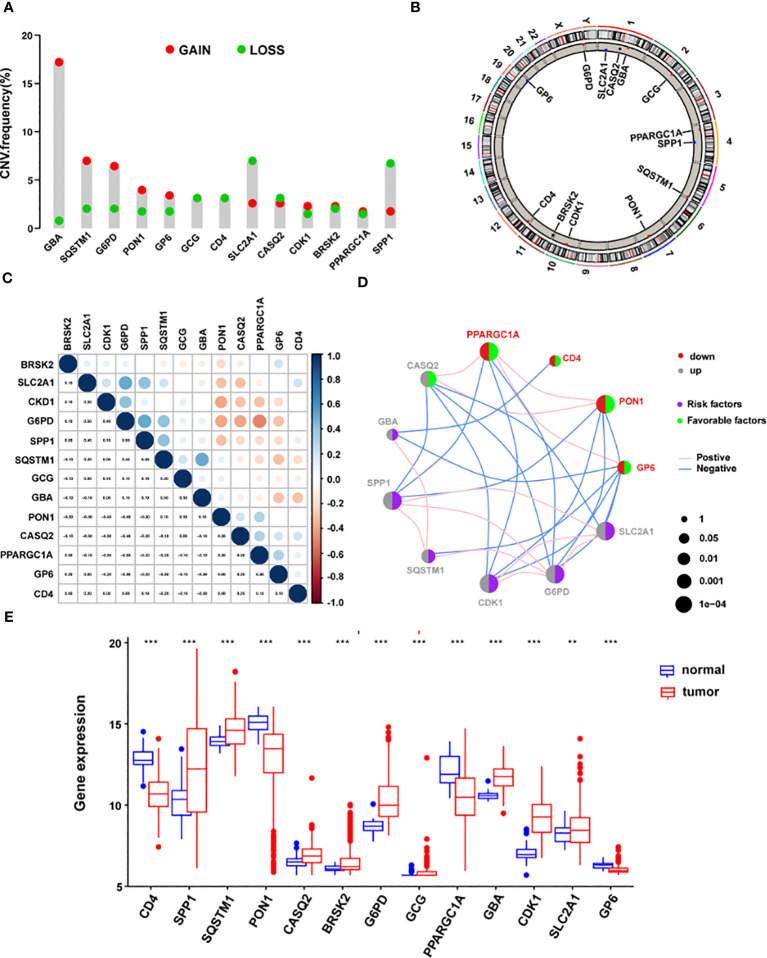
The landscape of genetic mutations of the signature genes in HCC. **(A)** The CNV alteration frequency of 13 ERS-related signature genes. The alteration frequency was represented by the column. A pink dot indicates the deletion frequency, whereas a blue dot indicates frequency. **(B)** The chromosomal location of CNVs in 13 ERS-related genes. **(C)** The relationship between the 13 ERS-associated signature genes. **(D)** Expression interactions on 13 ERS-related signature genes in HCC. **(E)** The mRNA levels of 13 ERS-related signature genes between LIHC and normal patients according to TCGA. *P < 0.05; **P < 0.01; ***P < 0.001. HCC, hepatocellular carcinoma; CNV, copy number variation; ERS, endoplasmic reticulum stress; LIHC, Liver Hepatocellular Carcinoma; TCGA, The Cancer Genome Atlas.

Subsequent examination of the hallmark genes indicated that CNV mutations were common. The expression of most ERS-related genes with CNV amplification, such as GBA, SQSTM1, G6PD, PON1, GP6, CDK1, BRSK2, and PPARGC1A, was considerably higher in TCGA-LIHC samples compared to normal control samples. SLC2A1, CASQ2, and SPP1 were downregulated in TCGA-LIHC samples simultaneously (**Figure 7A**). **Figure 7B** depicts the chromosomal locations of CNV changes in 13 ERS-related genes. In addition, the Spearman correlation was utilized to assess the reciprocal regulation of the signature genes (**Figure 7C**). The findings revealed that CASQ2, PPARGC1A, CD4, PON1, and GP6 were protective variables linked with longer OS, whereas GBA, SPP1, SQSTM1, CDK1, G6PD, and SLC2A1 were risk factors (**Figure 7D**). Additional study revealed that in tumor samples, CD4, PON1, PPARGC1A, and GP6 were dramatically downregulated, whereas SPP1, SQSTM1, CASQ2, GBA, CDK1, G6PD, BRSK2, GCG, and SLC2A1 were significantly elevated (all P < 0.05) (**Figure 7E**).

Additional survival studies were performed on the 13 signature genes in the TCGA-LIHC cohort to confirm their predictive significance. As a consequence, high levels of CD4, PON1, CASQ2, PPARGC1A, and GP6 expression were found to be substantially linked with extended survival time (all P < 0.05) (**Figures S4A, D, E, I, M**). Also, increased expression of SPP1, SQSTM1, G6PD, GBA, CDK1, SLC2A1, and was associated with a worse prognosis (all P < 0.05) (**Figures S4B, C, G, J, K, L**). Surprisingly, greater CASQ2 expression was linked to a prolonged survival duration (**Figure S4E**). No study has been conducted on the relationship between GP6 and, CASQ2, and HCC, despite these genes being strongly associated with prognosis. To investigate the mRNA levels of GP6 and CASQ2 in HCC specimens and cells, nine matched samples of HCC tissues and normal liver samples were examined using qRT-PCR. We found that only GP6 mRNA levels were notably reduced in HCC tissues than in matched normal samples (**Figures S5A, C**). Furthermore, we examined the expression of GP6 and CASQ2 in HCC cells and five noncancerous liver cell lines using qRT-PCR. Our results indicated that only GP6 mRNA levels were notably reduced in HCC cells compared to normal control (P < 0.05) (**Figures S5A, B**). Our analysis revealed significant differences and associations in the genomic and transcriptome landscapes of hallmark genes between normal and HCC samples. Specifically, alterations in the levels and genetic variation of ERS-related genes appeared to serve crucial roles in the control of HCC tumor progression.

### The role of the ERS-related score on the prediction of immunotherapeutic benefits

**Figure 8:**
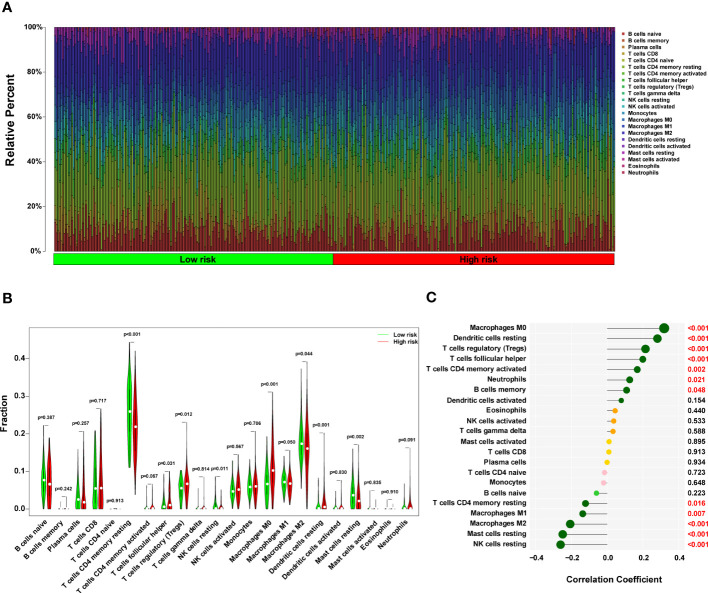
The immune landscape in the high-risk HCC patients. **(A)** The proportional percentage of infiltrating immune cells in individual samples is presented visually in a bar plot. **(B)** This violin plot shows the differentially infiltrated fraction of immune cells according to CIBERSORT. **(C)** The correlation between ERS-related score and infiltrated immune cells. HCC, hepatocellular carcinoma; ERS, endoplasmic reticulum stress.

A histogram of the general distribution of different immune cells in each sample is presented in **Figure 8A**, where the different hues represent numerous types of immune cells, and the height of each color represents the amount of that particular immune cell type in the sample. The CIBERSORT approach has limitations in revealing the location of immune cell subsets with low abundance in tumors. However, we observed individual differences in the proportion of immune cells between high- and low-risk groups. Cluster analyses of the immune cells infiltrated in disease and normal data are critical for identifying pathogenic processes and immunoregulatory systems. Resting Dendritic cells, Macrophages M0 cells, and T cells follicular helper infiltrated more in high-risk HCC patients (all P < 0.05), as seen in **Figure 8B**. On the other hand, resting T cells, CD4 memory cells, Mast cells resting cells, NK cells resting cells, and Macrophages M2 cells infiltrated less in high-risk HCC patients (all P < 0.05). In the high-risk group, NK resting cells penetrated the tissue less often as well (P < 0.05). In addition, a strong positive correlation was discovered between the risk score and M0 macrophages cells (r = 0.31, P = 1.33 x 10^-9^), resting Dendritic cells (r = 0.27, P = 2.45 x 10^-7^), T cells regulatory (Tregs) cells (r = 0.21, P = 6.5 x 10^-5^), T cells follicular helper (r = 0.19, P = 0.00020), T cells CD4 memory activated cells (r = 0.16, P = 0.0018), and Neutrophils cells (r = 0.12, P = 0.0208) (**Figure 8C** in addition to **Figure S6**). According to these findings, the group considered to be at high risk had a much more significant proportion of immune cells that had been improperly activated when compared to the group that was considered to be at low risk.

### A personalized prognostic prediction model

**Figure 9:**
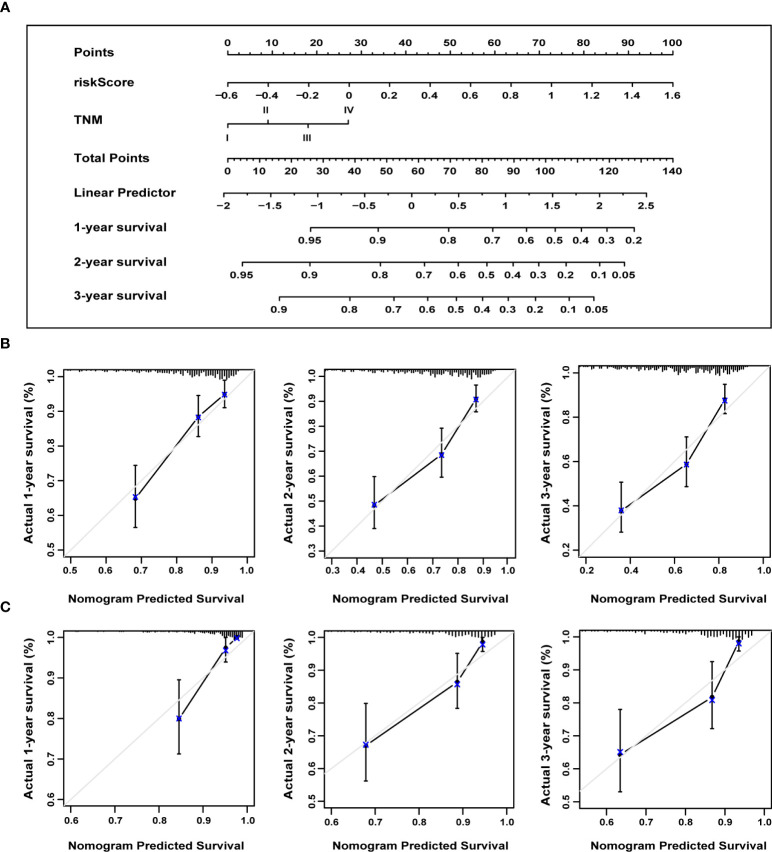
The nomogram to anticipate prognostic probabilities. **(A)** A nomogram incorporates the ERS-associated gene signature and the TNM stage. An analysis of the calibration curves for the nomograms predicting OS at 1, 2, and 3 years for the training **(B)** and validation cohorts **(C)**. ERS, endoplasmic reticulum stress; TNM, tumor-node-metastasis; OS, overall survival.

We constructed a nomogram to predict the probability of 1-, 2-, and 3-year OS using the 13-ERS-gene signature and TNM stage as predictors. As shown in **Figure 9A**, each component was assigned points based on its risk contribution to survival. According to calibration curves, actual and anticipated survival matched well (**Figure 9B**). Cindex values for the TNM model, prognostic gene signature, and nomogram model were 0.573, 0.729, and 0.731, respectively. For instance, if a patient had stage TNM III (18 points) and a high-risk score(80 points), she would be awarded 219 points. Her survival rates at 1, 2, and 3 years were approximately 55%, 30%, and 18%, respectively. We validated the nomogram using the validation cohort and plotted the calibration curves for 1-year, 2-year, and 3-year OS predictions, as illustrated in **Figure 9C**. Integrating our prognostic model with the standard TNM model may improve its predicting sensitivity and specificity, aiding clinical treatment choices.

### Discussion

HCC patients are at high risk of recurrence and mortality, and current staging systems may not reliably predict OS as they do not consider genetic and epigenetic factors. Therefore, there is a need for prognostic biomarkers that can identify HCC patients at higher risk of recurrence and poor survival who might benefit from more aggressive therapy. Recently, gene signatures based on specific characteristics have become a research focus for predicting cancer mortality risk, including immune, cell cycle, and other signatures. However, a global expression pattern based on ERS-associated genes has not been previously established in HCC. In this study, we developed a novel 13-ERS-gene signature that demonstrated excellent prognostic value in the TCGA-LIHC cohort and was validated in an independent ICGC LIRI-JP cohort. The 13-ERS-gene signature effectively stratified HCC patients into high and low-risk groups according to the 5-year OS, with an AUC value of 0.749 in the validation cohort. The discriminating performance of the 13-ERS-gene signature was statistically significant and clinically moderate, surpassing that of the TNM staging method alone. Therefore, the 13-ERS-gene signature could represent a novel genetic and epigenetic tool for prognosis prediction in HCC patients. Patients with HCC who have higher risk scores, as determined by the 13-ERS-gene signature, may benefit from more aggressive therapy and closer monitoring, which could potentially prolong their survival. Additionally, GSEA suggested that the 13 ERS-related genes may serve critical functions in the regulation of the cell cycle, spliceosome, homologous recombination, PPAR, drug metabolism cytochrome P450, adipocytokine, renin-angiotensin system, insulin signaling, and many metabolic pathways, shedding light on these genes’ biological functions.

Various intrinsic and microenvironmental disturbances could induce ERS in cancer cells, resulting in accumulated unfolded proteins and then the activation of UPR (12). The UPR functions in the re-establishment of homeostasis by reducing protein synthesis, inhibiting protein translation, facilitating misfolded proteins’ degradation, and regulating ERS-associated genes (16). ERS-associated genes and signalings can affect the pathophysiology of HCC, and previous studies have indicated its role in prognosis prediction (17). The platelet-specific collagen receptor GP6 causes platelet activation and controls various processes, such as platelet adhesion, aggregation, and procoagulant activity (18). Platelets aid in the growth and migration of tumors by stimulating the formation of blood and lymphatic vessels and shielding cancer cells from the innate immune system (19). Future study is required to clarify the functions of GP6 with ERS on the formation of HCC, even though we discovered that GP6 was downregulated in HCC tissues by qRT-PCR and a protective gene for the prognostic prediction of patients with HCC based on the expression of the gene. It is important to note that there is need for more available data about protein levels of GP6 in HCC, and no immunohistochemistry analyses have been conducted. This implies that further study is required to explore the role of GP6 in HCC and its potential implications for the diagnosis and treatment of HCC.

The CASQ2 could prevent the activation of IRE1α by binding directly to the luminal domain (of the ERS sensor) in the junctional sarcoplasmic reticulum (20). Previous studies have shown that CASQ2 has a significant impact on several cellular processes associated with breast cancer, including the proliferation, migration and invasion (21). In a bioinformatics study, it served crucial roles in lymph node metastasis and was substantially correlated with survival in bladder cancer patients (22). The reduction of tumor burden was seen in a mouse model for HCC with the inhibition of IRE1α-endonuclease activity (23). Although we could not identify differential expression of the CASQ2 in HCC, further investigation is needed to elucidate its potential role in HCC. The PON1 protects against oxidative stress by engaging in the hydrolysis of active oxidized phospholipids and removing lipid hydroperoxides and H2O2 via its peroxidase-like activity, implying its role in chronic liver impairment (24). In the meantime, the PON1 has been identified as a putative serum biomarker for microvascular invasion in HCC, and its expression is negatively associated with vascular invasion (25). Combining PON1 and AFP improves the accuracy of diagnosis for invasion in vascular in HCC patients compared to each test alone (26).

The regulation of CD4 and CD8 coreceptor gene expression is crucial for the development of T lymphocytes (27). The induced selective apoptosis of CD4+ T cells may promote the development of HCC (28). The PPARG has been shown to mitigate ERS and inflammation by modulating the expression of the nuclear growth factor receptor (29). The role of PPARG in HCC patients has been found to suppress tumor growth, angiogenesis, and migration (30, 31). Additionally, the PPARGC1A has been associated with susceptibility to HCC in an eastern Chinese Han population (32). The GCG-like peptide-1 could protect against non-alcoholic fatty liver disease by inhibiting the ERS-related pathway (33). The GCG receptor agonists have been shown to counteract hepatocarcinogenesis through the cAMP-PKA-EGFR-STAT3 axis in a non-alcoholic mouse model, while GCG may promote hepatocarcinogenesis in patients suffering from non-alcoholic fatty liver disease (34, 35). The protein encoded by the GBA3 is an intracellular enzyme that can catalyze the hydrolysis of various glycosides. The precise function of GBA3 in ERS remains uncertain. A strong correlation exists between reduced GBA3 expression and poor prognosis in individuals with HCC (36). The SPP1 appears to be involved in maintaining intracellular sphingosine-1-phosphate homeostasis, and its depletion leads to increased ERS and subsequent induction of autophagy through the UPR pathway (37). An elevated level of SPP1 was found in HCC, contributing to the progression (38).

The SQSTM1, also known as ubiquitin-binding protein p62, is involved in the degradation of misfolded proteins via selective autophagy (39). Perturbation of p62 activity has been observed in HCC, and it has been identified as a critical component of protein aggregates in the form of intracellular inclusion bodies. These inclusion bodies containing p62, are increasingly recognized as biomarkers for HCC (40). Moreover, p62 serves as a signaling scaffold involved in various physiological processes, including inflammation and programmed cell death. In HCC, dysregulation of these processes can contribute to tumor growth, invasion, and metastasis. p62 has also been linked to oxidative stress response and metabolism, which are important factors in HCC pathogenesis (40). CDK1 has been found to be involved in the regulation of ERS and its downstream signaling pathways. Previous studies have shown that the activation of UPR-associated transcription factors, including activating transcription factor 4 and C/EBP homologous protein, necessitates the presence of CDK1 activity (41). The CDK1-dependent phosphorylation of human telomerase reverse transcriptase promotes tumor growth in a telomere-independent manner (42). Moreover, the inhibition of cell proliferation in HCC was achieved by the downregulation of CDK1 (43). The therapeutic potential of CDKs in HCC has been demonstrated through their targeting of CDK signaling pathway (44). The role of BRSK2 in ERS appears to be related to the regulation of apoptosis. The downregulation of BRSK2 expression amplifies the ERS-induced apoptosis in cells. Additionally, the BRSK2 expression has been shown to modulate the mRNA levels of C/EBP homologous protein and cleaved caspase-3 (45). Further research would be necessary to determine any potential connection between BRSK2 and HCC. The inhibition of G6PD could induce ERS and its associated autophagy deregulation in breast cancer (46). G6PD overexpression has been shown to promote HCC cell motility and invasion by triggering epithelial-mesenchymal transition via the signal transducer and transcription activator three pathway (47). Knockdown of T-cell leukemia/lymphoma protein 1 increases the sensitivity of HCC to sorafenib, whereas knockdown of G6PD inhibits hepatocarcinogenesis (48). The expression of SLC2A1 is elevated in HCC and facilitates tumorigenesis (47). Moreover, long non-coding RNA SLC2A1-AS1 is involved in regulating aerobic glycolysis and progression in HCC through the STAT3/FOXM1/GLUT1 pathway (49). Most of the genes in this ERS-associated gene signature are involved in HCC progression, including proliferation, migration, invasion, and apoptosis. These processes have been demonstrated with ERS in some way. However, the research found no evidence of a regulatory relationship between these genes and the ERS pathway, such as the IRE1α, PERK, and ATF6. Further investigation is needed to elucidate their underlying mechanisms with the ERS pathway.

In the present research, we generated a nomogram that integrates the 13-gene ERS signature and the TNM stage to predict the 1-, 2-, and 3-year survival in HCC patients. Nomograms are broadly used in clinical practice due to their straightforward visual display. This is the first nomogram constructed and validated using large databases with long-term follow-up to predict the survival rate in HCC patients by combining an ERS-associated gene signature and the TNM stage. According to calibration plots based on the TCGA and ICGC databases, the nomogram had good predictive performance, as seen by how closely the actual survival matched the expected survival. This visual rating system might help physicians and patients make personalized survival forecasts, allowing them to choose better treatment alternatives.

The TME consists of complex interactions among stromal cells, tumor cells, and infiltrating immune cells, which could shape the course of HCC and predict the response and resistance to immunotherapies. In the present study, regulatory T cells were lower in the low-risk group, which is associated with worse OS. The role of regulatory T cells in the prognosis of HCC was well-described in the systematic review and meta-analysis (50). High infiltration rates of regulatory T cells predicted worse OS in 1214 patients. Furthermore, tumor-infiltrating neutrophils could stimulate the invasion of macrophages and Tregs into the TME via the production of monocyte chemotactic protein 1, leading to the progression of HCC and resistance to sorafenib (51). Targeting regulatory T cells and inhibiting the associated mediated molecules and pathways might inhibit HCC progression.

Although the ERS-associated gene signature showed promising results for survival prediction in HCC patients, several limitations must be considered. Firstly, experimental studies are necessary to understand the underlying mechanisms of these ERS-associated genes. Notably, there is an absence of available data regarding GP6 protein levels in HCC, and no immunohistochemistry analyses have been performed. Secondly, the signature was derived from the TCGA database and needs to be validated by other postoperative factors besides the TNM stage, such as the size of the tumor, number of tumor, Child-Pugh classification, and interval recurrence. Furthermore, the raw data does not adequately address other risk factors for HCC, such as metabolic disorders, race, diabetes, and smoking. Thirdly, prospective, observational, and multi-center studies are required to validate this predictive signature before its routine application. Additional investigation is required to elucidate the underlying mechanisms and establish a direct link between ERS-related genes and the TME in HCC.

## Conclusion

In conclusion, we created a unique and powerful 13-gene ERS signature that outperformed the traditional TNM stage in predicting prognosis in HCC patients. This signature efficiently separated the high-risk group of patients with HCC from the low-risk group. Dysregulated ERS signaling is present in many malignancies, and it aids in tumor survival by regulating other disease-related processes such as angiogenesis, transformation, and migration. Therefore, this signature has the potential to facilitate individualized treatment and achieve better outcomes for HCC patients. Moreover, GP6 inhibition might be a promising therapeutic method for HCC among these signature genes.

## Data availability statement

The original contributions presented in the study are included in the article/**Supplementary Material**. Further inquiries can be directed to the corresponding author.

## Ethics statement

The studies involving humans were approved by Ethics Committee of the First Affiliated Hospital of Chongqing Medical University. The studies were conducted in accordance with the local legislation and institutional requirements. The participants provided their written informed consent to participate in this study.

## Author contributions

KZ: Data curation, Formal Analysis, Investigation, Methodology, Writing – original draft. XY: Data curation, Investigation, Writing – review & editing. SL: Data curation, Writing – review & editing. YB: Conceptualization, Data curation, Formal Analysis, Writing – original draft.
