# Supplementary material for: Correlation of endoplasmic reticulum stress patterns with the immune microenvironment in hepatocellular carcinoma: a prognostic signature analysis
Source: Front Immunol. 2023 Dec 8;14:1270774. doi: 10.3389/fimmu.2023.1270774 (PMC10748430; doi:10.3389/fimmu.2023.1270774)
Supplement: Supplementary file 1 [file DataSheet_1.docx]

**Table SI. Characteristics of TCGA and ICGC HCC cohorts.**

| Characteristics | TCGA-LIHC cohort | | |  |
| --- | --- | --- | --- | --- |
|  | Total (n=364) | High risk (n=182) | Low risk (n=182) | P-value |
| Gender |  |  |  |  |
| Famale | 119 (32.7) | 62 (34.1) | 57 (31.3) | 0.655 |
| Male | 245 (67.3) | 120 (65.9) | 125 (68.7) |  |
| Age (years) |  |  |  |  |
| <60 | 165 (45.3) | 80 (44.0) | 85 (46.7) | 0.674 |
| ≥60 | 199 (54.7) | 102 (56.0) | 97 (53.3) |  |
| Tumor grade |  |  |  |  |
| G1 | 55 (15.1) | 16 (8.8) | 39 (21.4) | <0.001 |
| G2 | 179 (49.2) | 80 (44.0) | 99 (54.4) |  |
| G3 | 118 (32.4) | 78 (42.9) | 40 (22.0) |  |
| G4 | 12 (3.3) | 8 (4.4) | 4 (2.2) |  |
| T (Tumour) |  |  |  |  |
| T1 | 180 (49.5) | 65 (35.7) | 115 (63.2) | <0.001 |
| T2 | 93 (25.5) | 53 (29.1) | 40 (22.0) |  |
| T3 | 78 (21.4) | 55 (30.2) | 23 (12.6) |  |
| T4 | 13 (3.6) | 9 (4.9) | 4 (2.2) |  |
| N (Lymph Node) |  |  |  |  |
| NO | 248 (68.1) | 128 (70.3) | 120 (65.9) | 0.431 |
| N1 | 116 (31.9) | 54 (29.7) | 62 (34.1) |  |
| M (Metastasis) |  |  |  |  |
| M0 | 262 (72.0) | 135 (74.2) | 127 (69.8) | 0.414 |
| M1 | 102 (28.0) | 47 (25.8) | 55 (30.2) |  |
| TNM stage |  |  |  |  |
| I | 112 (30.8) | 38 (20.9) | 74 (40.7) | <0.001 |
| II | 51 (14.0) | 32 (17.6) | 19 (10.4) |  |
| III | 62 (17.0) | 43 (23.6) | 19 (10.4) |  |
| IV | 139 (38.2) | 69 (37.9) | 70 (38.5) |  |
|  | ICGC LIRI-JP cohort | | |  |
|  | Total (n=231) | High risk (n=130) | Low risk (n=101) |  |
| Gender |  |  |  |  |
| Famale | 61 (26.4) | 34 (26.2) | 27 (26.7) | 1 |
| Male | 170 (73.6) | 96 (73.8) | 74 (73.3) |  |
| Age (years)  <60  ≥60 | 44 (19.0)  187 (81.0) | 23 (17.7)  107 (82.3) | 21 (20.8)  80 (79.2) | 0.67 |
| TNM stage |  |  |  |  |
| I | 36 (15.6) | 15 (11.5) | 21 (20.8) | 0.001 |
| II | 105 (45.5) | 50 (38.5) | 55 (54.5) |  |
| III | 71 (30.7) | 49 (37.7) | 22 (21.8) |  |
| IV | 19 (8.2) | 16 (12.3) | 3 (3.0) |  |

**Table SII. Univariate Cox regression analysis of ER stress genes associated with the survival of HCC.**

| **NO.** | **Gene Symbol** | **Hazard Ratio** | **P-value** |
| --- | --- | --- | --- |
| 1 | CAT | 0.779(0.667-0.91) | 0.002 |
| 2 | INS | 0.713(0.541-0.939) | 0.016 |
| 3 | CACNA1C | 0.774(0.614-0.977) | 0.031 |
| 4 | MAPT | 1.26(1.082-1.467) | 0.003 |
| 5 | EPM2A | 0.691(0.5-0.955) | 0.025 |
| 6 | HMOX1 | 1.158(1.007-1.333) | 0.04 |
| 7 | ALB | 0.911(0.85-0.978) | 0.01 |
| 8 | CDKN3 | 1.27(1.075-1.5) | 0.005 |
| 9 | KCNH2 | 1.189(1.044-1.354) | 0.009 |
| 10 | CD4 | 0.849(0.721-0.999) | 0.048 |
| 11 | SPP1 | 1.13(1.071-1.193) | <0.001 |
| 12 | SLC26A2 | 1.315(1.023-1.69) | 0.033 |
| 13 | F9 | 0.918(0.859-0.981) | 0.012 |
| 14 | GLA | 1.242(1.015-1.521) | 0.036 |
| 15 | SEC24B | 0.792(0.629-0.997) | 0.047 |
| 16 | KCNJ11 | 1.271(1.068-1.512) | 0.007 |
| 17 | SERPINA3 | 0.857(0.759-0.967) | 0.012 |
| 18 | APOA1 | 0.919(0.867-0.975) | 0.005 |
| 19 | C1S | 0.789(0.695-0.895) | <0.001 |
| 20 | C1R | 0.823(0.717-0.944) | 0.005 |
| 21 | ATF3 | 0.841(0.708-0.999) | 0.049 |
| 22 | HP | 0.932(0.875-0.992) | 0.026 |
| 23 | XDH | 0.888(0.808-0.976) | 0.013 |
| 24 | SQSTM1 | 1.284(1.087-1.518) | 0.003 |
| 25 | CKAP4 | 1.264(1.036-1.542) | 0.021 |
| 26 | CACNA1S | 1.793(1.049-3.064) | 0.033 |
| 27 | PON1 | 0.858(0.802-0.918) | <0.001 |
| 28 | CASQ2 | 0.585(0.44-0.779) | <0.001 |
| 29 | IGF2BP2 | 1.121(1.009-1.246) | 0.033 |
| 30 | BRSK2 | 1.249(1.05-1.487) | 0.012 |
| 31 | SGK1 | 0.851(0.743-0.975) | 0.02 |
| 32 | ESR1 | 0.799(0.698-0.914) | 0.001 |
| 33 | G6PC | 0.866(0.807-0.928) | <0.001 |
| 34 | LDLR | 0.753(0.619-0.917) | 0.005 |
| 35 | NQO1 | 1.086(1.023-1.152) | 0.007 |
| 36 | G6PD | 1.395(1.242-1.566) | <0.001 |
| 37 | EGF | 1.223(1.052-1.422) | 0.009 |
| 38 | GABARAPL1 | 0.766(0.66-0.888) | <0.001 |
| 39 | DCN | 0.919(0.849-0.996) | 0.04 |
| 40 | NR3C2 | 0.766(0.626-0.937) | 0.01 |
| 41 | IGF1 | 0.826(0.719-0.95) | 0.007 |
| 42 | BSG | 1.204(1.007-1.44) | 0.041 |
| 43 | FOXO1 | 0.667(0.535-0.831) | <0.001 |
| 44 | AGR2 | 1.095(1.02-1.176) | 0.012 |
| 45 | MATN3 | 1.172(1.04-1.32) | 0.009 |
| 46 | GCG | 1.257(1.003-1.575) | 0.047 |
| 47 | PPARGC1A | 0.81(0.736-0.892) | <0.001 |
| 48 | MMP9 | 1.108(1.002-1.224) | 0.045 |
| 49 | GBA | 1.277(1.013-1.61) | 0.039 |
| 50 | CDK1 | 1.368(1.173-1.597) | <0.001 |
| 51 | ACAN | 1.188(1.003-1.407) | 0.046 |
| 52 | TTR | 0.914(0.855-0.978) | 0.009 |
| 53 | SCN4A | 0.617(0.463-0.823) | 0.001 |
| 54 | SLC2A1 | 1.423(1.233-1.643) | <0.001 |
| 55 | MYH6 | 2.407(1.451-3.991) | 0.001 |
| 56 | TTPA | 0.892(0.808-0.985) | 0.024 |
| 57 | GP6 | 0.265(0.115-0.61) | 0.002 |
| 58 | QDPR | 0.767(0.649-0.908) | 0.002 |
| 59 | SGPP2 | 1.224(1.073-1.395) | 0.003 |

**Table SIII.** **Gene set enrichment analyses between high and low risk group in TCGA-LIHC and** **ICGC LIRI-JP cohorts.**

**C2 KEGG Terms enriched in high risk group**

| **NAME** | **SIZE** | **ES** | | | **NES** | **NOM p-val** | **FDR q-val** | |
| --- | --- | --- | --- | --- | --- | --- | --- | --- |
| **TCGA-LIHC (2 terms)** |  | |  |  | | |  |  |
| KEGG_HOMOLOGOUS_RECOMBINATION | 28 | | 0.673 | 1.714 | | | 0.008 | 0.182 |
| KEGG_SPLICEOSOME | 126 | | 0.494 | 1.702 | | | 0.049 | 0.141 |
| **ICGC LIRI-JP (1 terms)** |  | |  |  | | |  |  |
| KEGG_CELL_CYCLE | 124 | | 0.639 | 1.718 | | | 0.008 | 0.223 |
|  |  | |  |  | | |  |  |
| **C2 KEGG Terms enriched in low risk group** |  | |  |  | | |  |  |
| **NAME** | **SIZE** | | **ES** | **NES** | | | **NOM p-val** | **FDR q-val** |
| **TCGA-LIHC (15 terms)** |  | |  |  | | |  |  |
| KEGG_PPAR_SIGNALING_PATHWAY | 69 | | -0.716 | -1.568 | | | 0.004 | 0.246 |
| KEGG_BETA_ALANINE_METABOLISM | 22 | | -0.765 | -1.563 | | | 0.014 | 0.236 |
| KEGG_CYSTEINE_AND_METHIONINE_METABOLISM | 32 | | -0.663 | -1.555 | | | 0.020 | 0.231 |
| KEGG_GLYCINE_SERINE_AND_THREONINE_METABOLISM | 31 | | -0.837 | -1.554 | | | 0.006 | 0.216 |
| KEGG_ADIPOCYTOKINE_SIGNALING_PATHWAY | 66 | | -0.558 | -1.548 | | | 0.016 | 0.210 |
| KEGG_DRUG_METABOLISM_CYTOCHROME_P450 | 70 | | -0.774 | -1.531 | | | 0.006 | 0.211 |
| KEGG_RENIN_ANGIOTENSIN_SYSTEM | 16 | | -0.747 | -1.522 | | | 0.024 | 0.213 |
| KEGG_TYROSINE_METABOLISM | 42 | | -0.674 | -1.510 | | | 0.020 | 0.222 |
| KEGG_RETINOL_METABOLISM | 64 | | -0.784 | -1.504 | | | 0.018 | 0.219 |
| KEGG_PRIMARY_BILE_ACID_BIOSYNTHESIS | 16 | | -0.892 | -1.496 | | | 0.012 | 0.221 |
| KEGG_BUTANOATE_METABOLISM | 34 | | -0.700 | -1.490 | | | 0.048 | 0.221 |
| KEGG_LINOLEIC_ACID_METABOLISM | 28 | | -0.725 | -1.484 | | | 0.036 | 0.210 |
| KEGG_GLYCEROLIPID_METABOLISM | 48 | | -0.502 | -1.484 | | | 0.020 | 0.202 |
| KEGG_ARGININE_AND_PROLINE_METABOLISM | 53 | | -0.628 | -1.480 | | | 0.038 | 0.199 |
| KEGG_GLYOXYLATE_AND_DICARBOXYLATE_METABOLISM | 15 | | -0.681 | -1.479 | | | 0.048 | 0.193 |
| **ICGC LIRI-JP (15 terms)** |  | |  |  | | |  |  |
| KEGG_INSULIN_SIGNALING_PATHWAY | 137 | | -0.440 | -1.542 | | | 0.014 | 0.238 |
| KEGG_GLYCEROLIPID_METABOLISM | 43 | | -0.549 | -1.541 | | | 0.014 | 0.221 |
| KEGG_GLYOXYLATE_AND_DICARBOXYLATE_METABOLISM | 16 | | -0.749 | -1.540 | | | 0.008 | 0.205 |
| KEGG_BETA_ALANINE_METABOLISM | 22 | | -0.791 | -1.540 | | | 0.027 | 0.192 |
| KEGG_FATTY_ACID_METABOLISM | 42 | | -0.849 | -1.533 | | | 0.004 | 0.191 |
| KEGG_HISTIDINE_METABOLISM | 27 | | -0.745 | -1.518 | | | 0.018 | 0.204 |
| KEGG_ARGININE_AND_PROLINE_METABOLISM | 51 | | -0.684 | -1.510 | | | 0.043 | 0.206 |
| KEGG_LINOLEIC_ACID_METABOLISM | 29 | | -0.825 | -1.495 | | | 0.006 | 0.219 |
| KEGG_GLYCINE_SERINE_AND_THREONINE_METABOLISM | 31 | | -0.867 | -1.485 | | | 0.006 | 0.225 |
| KEGG_TYROSINE_METABOLISM | 40 | | -0.747 | -1.475 | | | 0.021 | 0.224 |
| KEGG_DRUG_METABOLISM_CYTOCHROME_P450 | 72 | | -0.825 | -1.458 | | | 0.006 | 0.224 |
| KEGG_RETINOL_METABOLISM | 63 | | -0.854 | -1.438 | | | 0.012 | 0.244 |


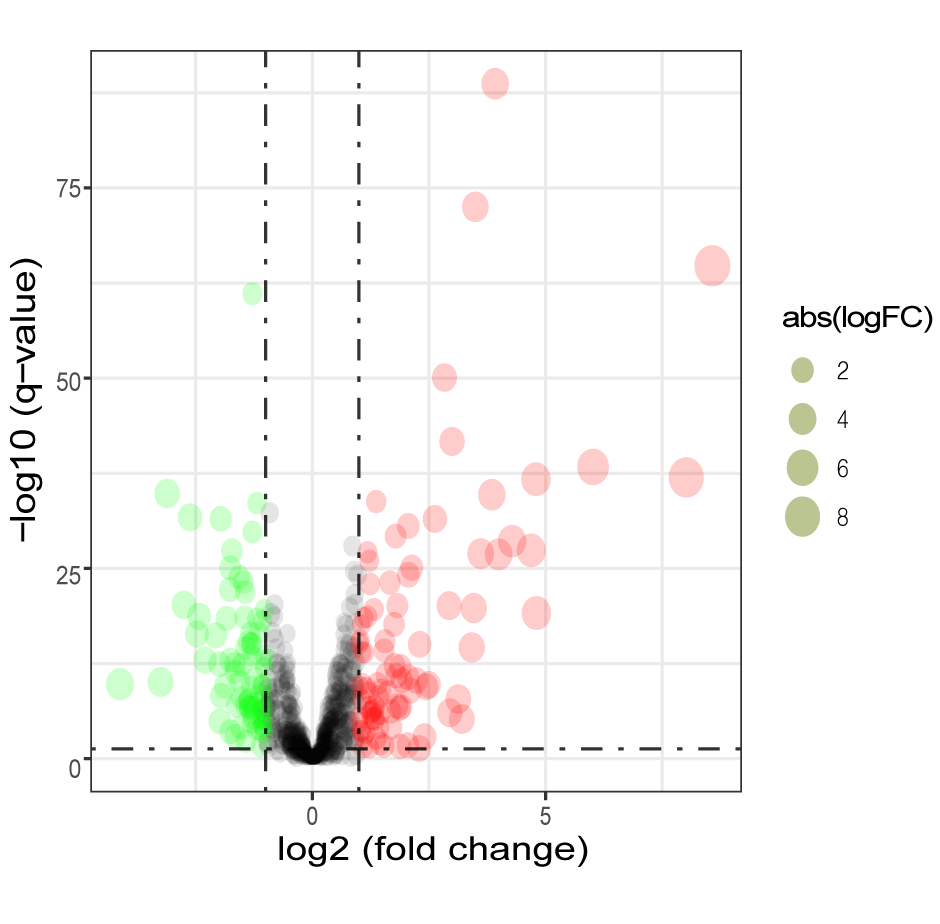


**Figure S1. Volcano plot of the differentially expressed ERS-related genes between HCC and non-tumor tissues.**Red dots indicated ERS-related genes which were high expression in HCC and blue for low expression. This volcano plot was drawn by the ggplot2 package of R language.


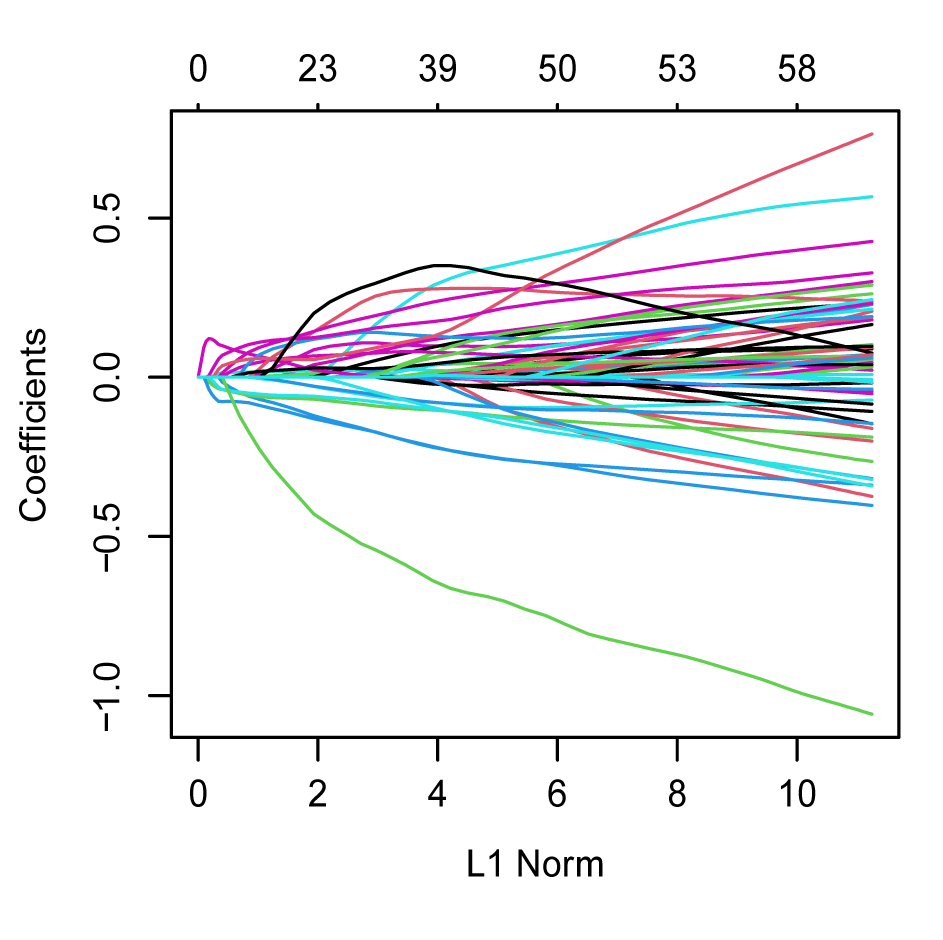


**Figure S2. LASSO coefficient profiles of the 59 ERS-related genes.**

**
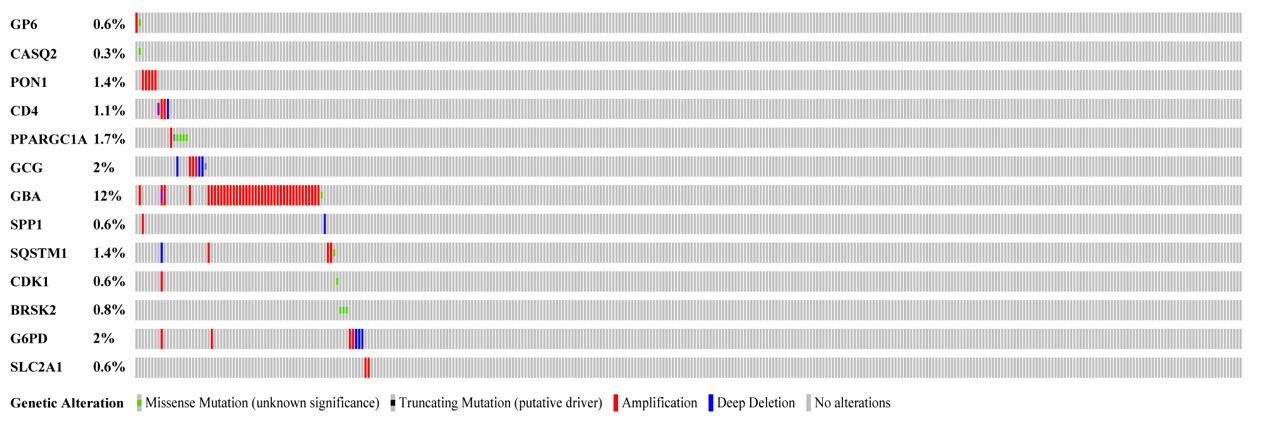
**

**Figure S3. Genetic alteration of genes included in the 13 ERS-related gene signature (TCGA, PanCancer Atlas).**

**
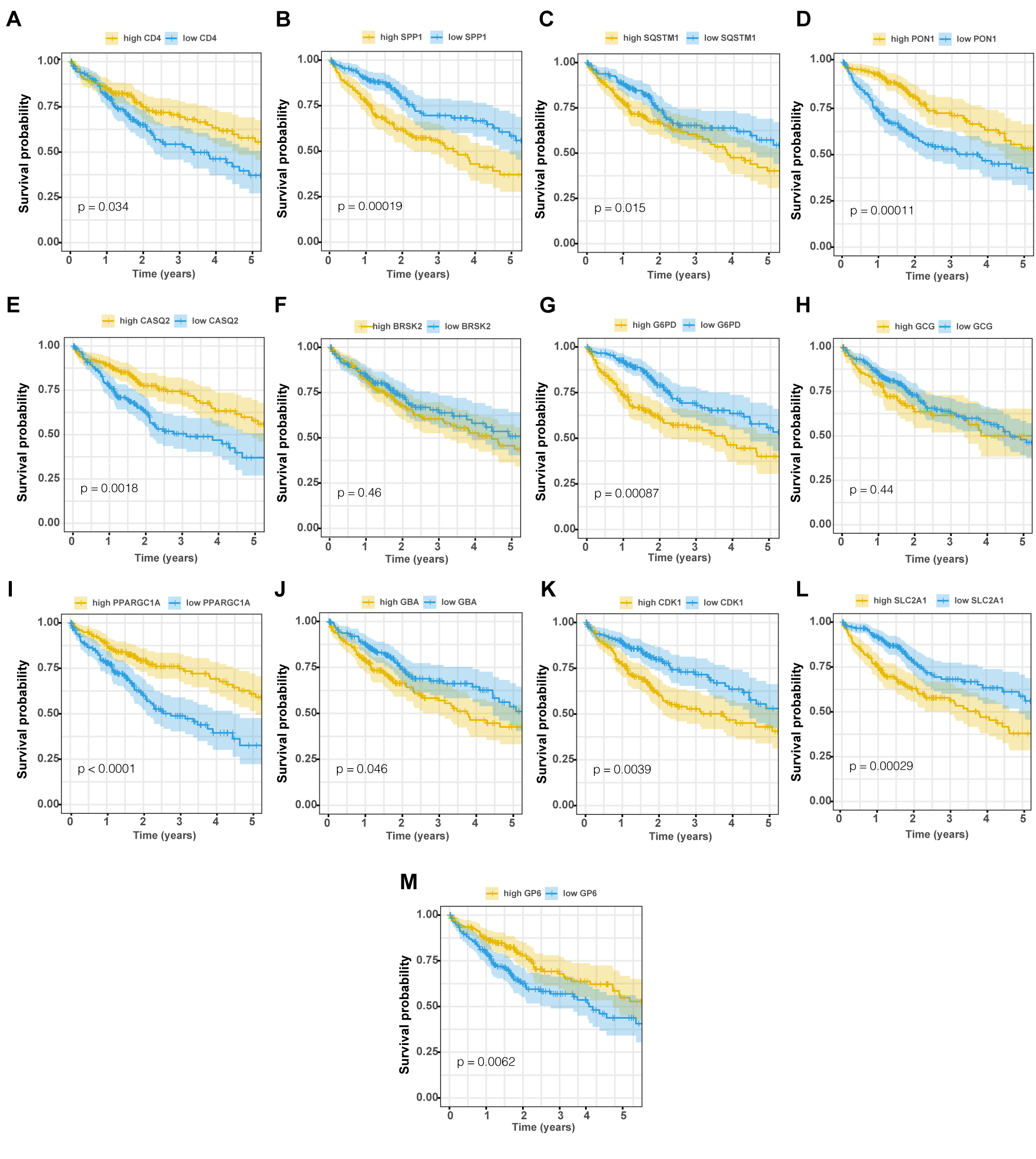
**

**Figure S4. The prognostic value of these ERS-related signature genes in HCC.**

**
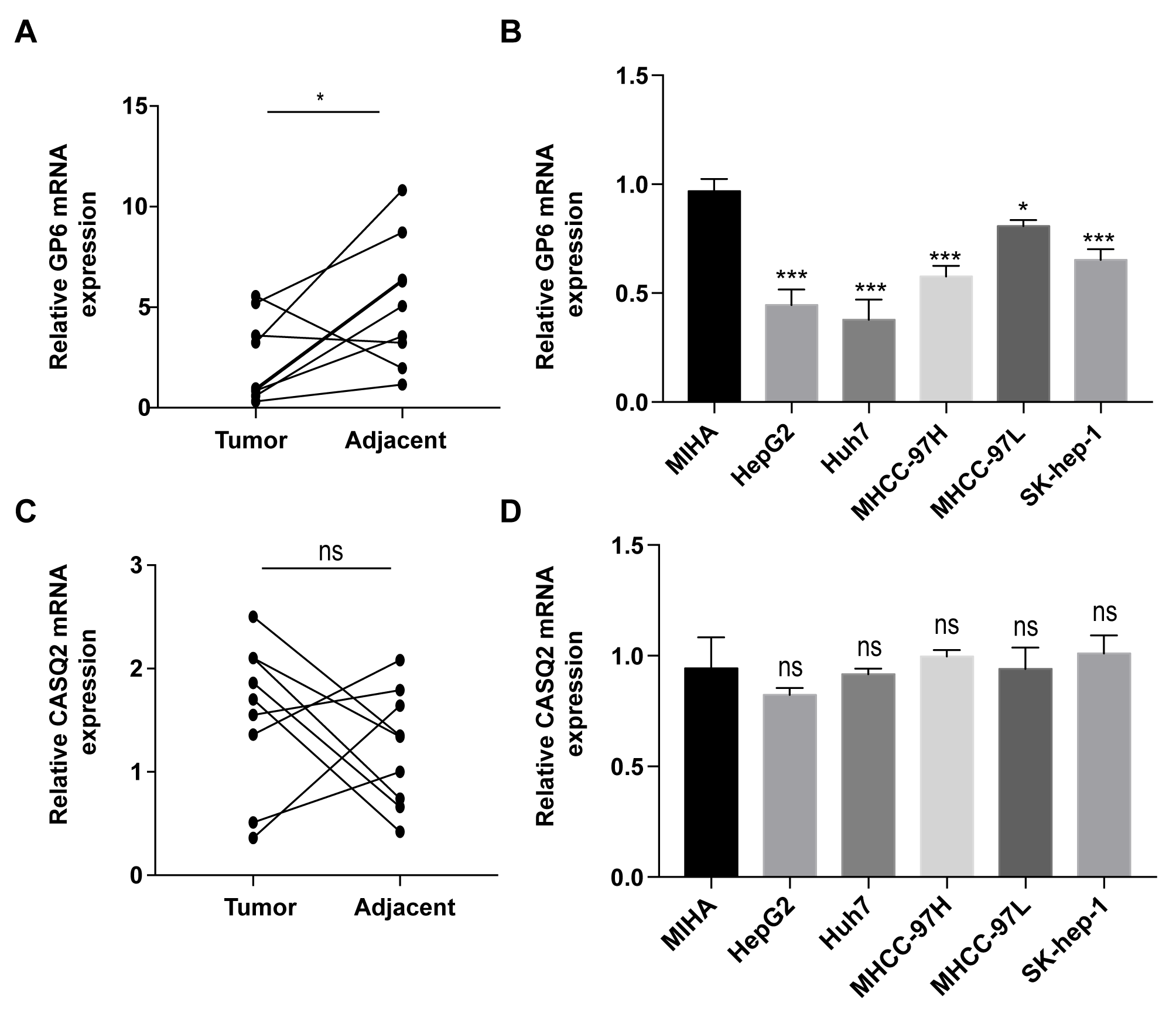
**

**Figure S5. The GP6 and CASQ2 mRNA expression in HCC.** (A) The mRNA expression GP6 in 9 pairs of HCC samples. (B) The mRNA expression of GP6 in noncancerous liver cell lines and five HCC cell lines. (C) The mRNA expression CASQ2 in 9 pairs of HCC samples. (D) The mRNA expression of CASQ2 in noncancerous liver cell lines and five HCC cell lines.


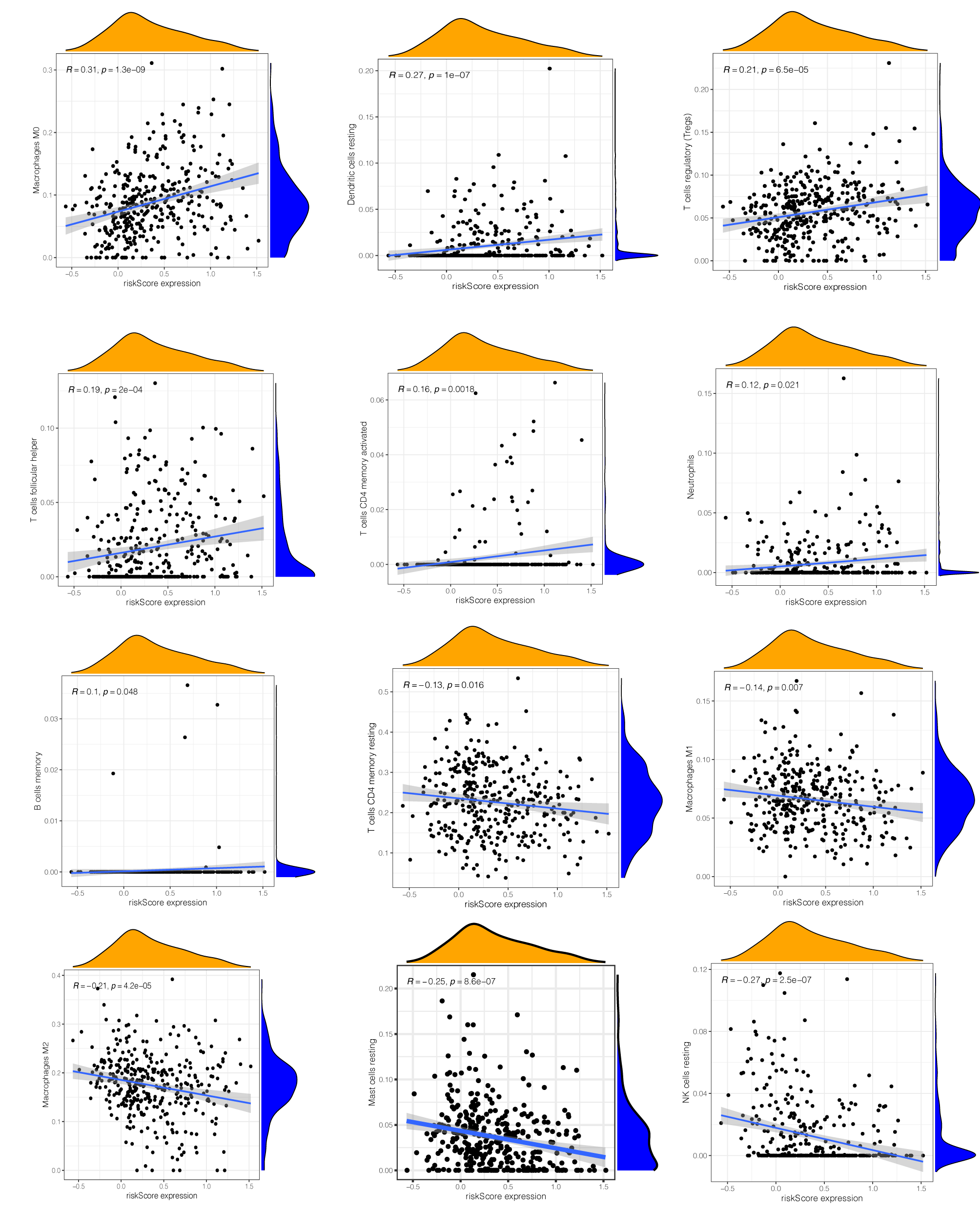


**Figure S6. The correlation of the ERS score and the immune cells.**
